# Evolution and global research trends of immunity in diabetic nephropathy: a bibliometric and visual analysis from 2004 to 2023

**DOI:** 10.1007/s11255-024-04081-x

**Published:** 2024-05-17

**Authors:** Jianlong Zhou, Lv Zhu, Rensong Yue

**Affiliations:** 1 Department of Clinical Medicine, People’s Hospital of Deyang City, Deyang, China; 2 West China Center of Excellence for Pancreatitis, Institute of Integrated Traditional Chinese and Western Medicine, West China Hospital, Sichuan University, Chengdu, China; 3 Department of Clinical Medicine, Hospital of Chengdu University of Traditional Chinese Medicine, Chengdu, China

**Keywords:** Immunity, Diabetic nephropathy, Bibliometric, Visual analysis, Research trends

## Abstract

**Background:**

Diabetic nephropathy (DN) is the leading cause of end-stage renal disease, with an increasing prevalence worldwide, but its pathomechanisms remain incompletely understood. Accumulating evidence suggests that immunity plays an important role in the development of DN. Many papers have been published in the field over the last 20 years, but there has been no bibliometric review of the research hotspots and trends in the field. This study aimed to assess the current research status and future trends of the link between immune and DN using bibliometric analysis.

**Methods:**

Publications on the association between immunity and DN from 2004 to 2023 were retrieved from the Web of Science Core Collection database and screened according to inclusion criteria. VOSviewer and CiteSpace software were employed to visualize research trends and hotspots in the field. Data including author, institution, country, journal, reference, and keyword were analyzed.

**Results:**

Ultimately 1246 publications meeting the criteria were included in the bibliometric analysis, involving 838 articles (84.96%) and 408 reviews (15.04%). The literature covered 81 countries and regions, 1751 institutions, and 6584 authors. The top 2 countries in terms of the number of publications were China (435) and the United States (318), and they collaborated most frequently. The United States had the highest number of citations for published papers (18,161), far exceeding the other countries. England had 38 publications but had the highest average number of citations (92.32). The University of California system was the most prolific institution (25 papers, 1062 citations, 42.48 citations per paper). *Frontiers in Immunology* was the most prolific journal in the field (30 papers). The most cited journal was *Kidney International* (863 citations). The analysis of keywords and references showed that inflammation, ferroptosis, and lipid metabolism may be future research hotspots in this field.

**Conclusions:**

The number of publications related to immunity and DN has increased annually over the past 20 years, with a significant increase in the last 3 years especially. Our results identified research hotspots and trends in the field. These findings provide valuable perspectives for future research, enhancing our understanding of the immune-related mechanisms of DN and exploring potential therapeutic strategies.

## Introduction

Diabetic nephropathy (DN), a common and serious microvascular complication of diabetes, is the leading cause of end-stage renal disease (ESRD) and is associated with increased risks of cardiovascular disease and death in high-risk individuals [1, 2]. The International Diabetes Federation (IDF) estimated that 537 million adults aged 20 to 79 years suffered from diabetes in 2021. The global number of patients with diabetes is projected to increase to 784 million by 2045 [3]. Up to 40% of people with diabetes will develop chronic kidney disease (CKD) [4]. According to a recent global burden of disease study, the incidence cases of CKD due to type 2 diabetes worldwide have increased by 74%, from 1.35 million cases in 1990 to 2.35 million cases in 2017 [5]. DN not only reduces the quality of life of diabetic patients but also imposes a serious economic burden on the family and society [6]. Hyperglycemia in diabetic patients has long been recognized as the initiating factor for the development of DN [7]. Renal hemodynamic impairment caused by hyperglycemia and disorders of glucose and lipid metabolism are the two major pathophysiologic bases of DN [8]. Based on these two points of view, the treatment of DN mainly focuses on the control of blood glucose, blood lipids, blood pressure, and the improvement of renal hemodynamics [9]. However, even if the control of blood glucose, blood lipids, and blood pressure is achieved, the development of DN cannot be completely prevented [10]. This suggests that there are other mechanisms involved in the development of DN in addition to the factors mentioned above. Furthermore, although renal replacement therapy (RRT) is the mainstay for patients with ESRD, its accessibility is severely lacking in low- and lower-middle-income countries [11, 12]. Unfortunately, the pathogenesis of DN is not fully understood [13]. Therefore, there is an urgent to improve the understanding of the pathogenesis of DN to diagnose it at an early stage and to discover new therapeutic agents.

The immune system consists of innate and adaptive immunity [14]. Immune cell infiltration is an important feature of DN [15]. There is growing evidence that immunity plays an important role in the pathogenesis of DN [16–19], which involves key members of the innate and adaptive immune systems [20–22]. The immune response involved in DN is mainly an intrinsic immune response [23]. Macrophages are the most common infiltrating cells in DN renal tissues and are associated with decreased renal function. Studies have shown that diabetes mellitus promotes the expression of Toll-like receptor 4 (TLR4) in macrophages and renal tubular cells, which induces inflammatory cytokines, such as interleukin-6 (IL-6), thereby causing amplification of renal tubulointerstitial inflammation and exacerbation of injury [24]. In renal biopsy tissues from patients with DN, macrophages were found to accumulate predominantly in renal tubules that underwent injury (e.g., around tubular dilatation, atrophy, and apoptotic cells) during the early stages of DN, which was significantly and positively correlated with the patient's serum creatinine and proteinuria levels as well as glomerulosclerosis and interstitial fibrosis [25]. Adaptive immune system component cells include helper (CD4 +) T cells, cytotoxic (CD8 +) T cells, and B cells. The development of kidney disease in diabetic patients is associated with activation of circulating T cells and increased T cells and C–C motif chemokine 5 (CCL5) in the kidney [26]. Besides, a study using single-cell RNA sequencing found that immune cell marker genes including EIF4B, RICTOR, and PRKCB were significantly higher expressed in diabetic kidney specimens than in controls, and experimentally validated that they may serve as potential therapeutic targets for DN [27]. Overall, these findings suggest a major role for immunity in the development of DN.

Emerging in 1969, bibliometrics is a complex analytical method that combines mathematics, statistics, and bibliography [28]. Bibliometric analysis differs from traditional systematic evaluation in that it allows for quantitative and qualitative assessment of publications. Bibliometric analysis and visualization of literature, authors, institutions, and countries/regions can help to understand the research hotspots and trends in a particular field over a certain period [29]. Several researchers have used bibliometrics to explore the relationship between intestinal microbiota and DN [30], mucosal immunity, and IGA nephropathy [31], as well as the immune system and osteoporosis [32]. Over the past two decades, more and more studies on the relationship between immunity and DN have begun to appear [16–19]. However, to the best of our knowledge, there is currently no published bibliometric analysis focusing on immunization and DN. Therefore, to gain a more comprehensive understanding of the evolution and trends in this field, we visualized various bibliometric indicators by combing the research results on immunization and DN during the past twenty years by using bibliometric analysis tools. We hope to provide valuable insights for future research through the construction of a scientific knowledge graph of the field.

## Materials and methods

### Data sources and search strategy

The Web of Science Core Collection (WoSCC) database (https://www.webofscience.com/wos/woscc/basic-search) has better accuracy in labeling literature types than any other database and is considered the best choice for literature analysis [33]. Therefore, we chose to conduct the retrieval in this database. On January 15, 2024, we searched the WOS database for all articles published between 2004 and December 31, 2023, related to the role of immunity in DN. The search strategy was as follows: ((((((TS = (immunity)) OR TS = (immunize)) OR TS = (immune)) OR TS = (immunization)) OR TS = (immunifaction))) OR TS = (immunothera*) AND (((((((((((((((((TS = (Diabetic Nephropathies)) OR TS = (Nephropathies, Diabetic)) OR TS = (Nephropathy, Diabetic)) OR TS = (Diabetic Nephropathy)) OR TS = (Diabetic Kidney Disease)) OR TS = (Diabetic Kidney Diseases)) OR TS = (Kidney Disease, Diabetic)) OR TS = (Kidney Diseases, Diabetic)) OR TS = (Diabetic Glomerulosclerosis)) OR TS = (Glomerulosclerosis, Diabetic)) OR TS = (Intracapillary Glomerulosclerosis)) OR TS = (Nodular Glomerulosclerosis)) OR TS = (Glomerulosclerosis, Nodular)) OR TS = (Kimmelstiel-Wilson Syndrome)) OR TS = (Kimmelstiel Wilson Syndrome)) OR TS = (Syndrome, Kimmelstiel-Wilson)) OR TS = (Kimmelstiel-Wilson Disease)) OR TS = (Kimmelstiel Wilson Disease).

### Study selection and data extraction

All relevant publications were independently assessed by two authors and each disagreement was fully discussed with a third author. Literature selection for this study was based on the following inclusion criteria: (1) there were full-text publications related to the role of immunity in DN; (2) the manuscripts of the articles and reviews were written in English; and (3) the literature was published from January 1, 2004, to December 31, 2023. The exclusion criteria were as follows: (1) the topic was not related to the role of immunization in DN; and (2) the type of article was a conference abstract, news, briefing paper, etc. Plain text versions of the publications were exported for analysis. The following data were extracted from the included publications: title, institution, country, journal, year of publication, number of citations, keywords, references, etc.

### Bibliometric and visualized analysis

GraphPad Prism v8.0.2 software was used to analyze trends and proportions of annual publications and national publications. In addition, CtieSpace (Premium version 6.2.4R (64-bit)) and VOSviewer (version 1.6.18) were used to analyze the extracted data and visualize the scientific knowledge graph. The VOSviewer v.1.6.17, created by Waltman et al. in 2009, is a free JAVA-based software for analyzing large amounts of literature data and displaying it as a map format [34]. In this study, VOSviewer software was used to create visual graphs and analyze the most prolific journals, author collaborations, and high frequency keywords. To visualize the results of research in a particular field by mapping the literature co-citation network, Professor Chaomei Chen created the CiteSpace (6.1.6R) software, which envisions the use of an experimental framework for studying new concepts and evaluating existing technologies [35]. This enables users to better understand areas of knowledge, research frontiers, and trends, and to predict their future research perspectives. This study used CiteSpace software to visualize country/institution collaborations, co-cited journals, co-cited authors, co-cited references, and keyword clustering.

This study was a bibliometric analysis of existing publications and did not require ethical approval.

## Results

### Literature search and characteristics

**Fig. 1:**
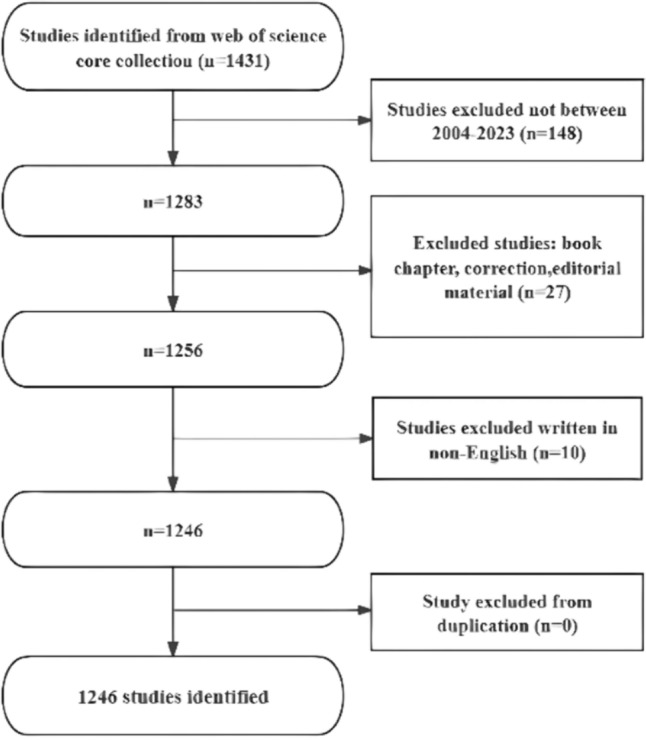
Flow diagram of the literature search

According to the search strategy, the WoSCC database contained 1431 publications on the role of immunity in DN. First, we excluded 148 studies that were not between 2004 and 2023. Then, 27 studies including book chapters, errata, and editorial material were excluded. Ultimately, after excluding 10 studies that were not written in English, 1246 studies meeting the inclusion criteria were included in the bibliometric analysis, including 838 (84.96%) articles and 408 reviews (15.04%). The specific literature search process is shown in Fig. 1. The literature covered 81 countries and regions, 1751 institutions, and 6584 authors.

**Fig. 2:**
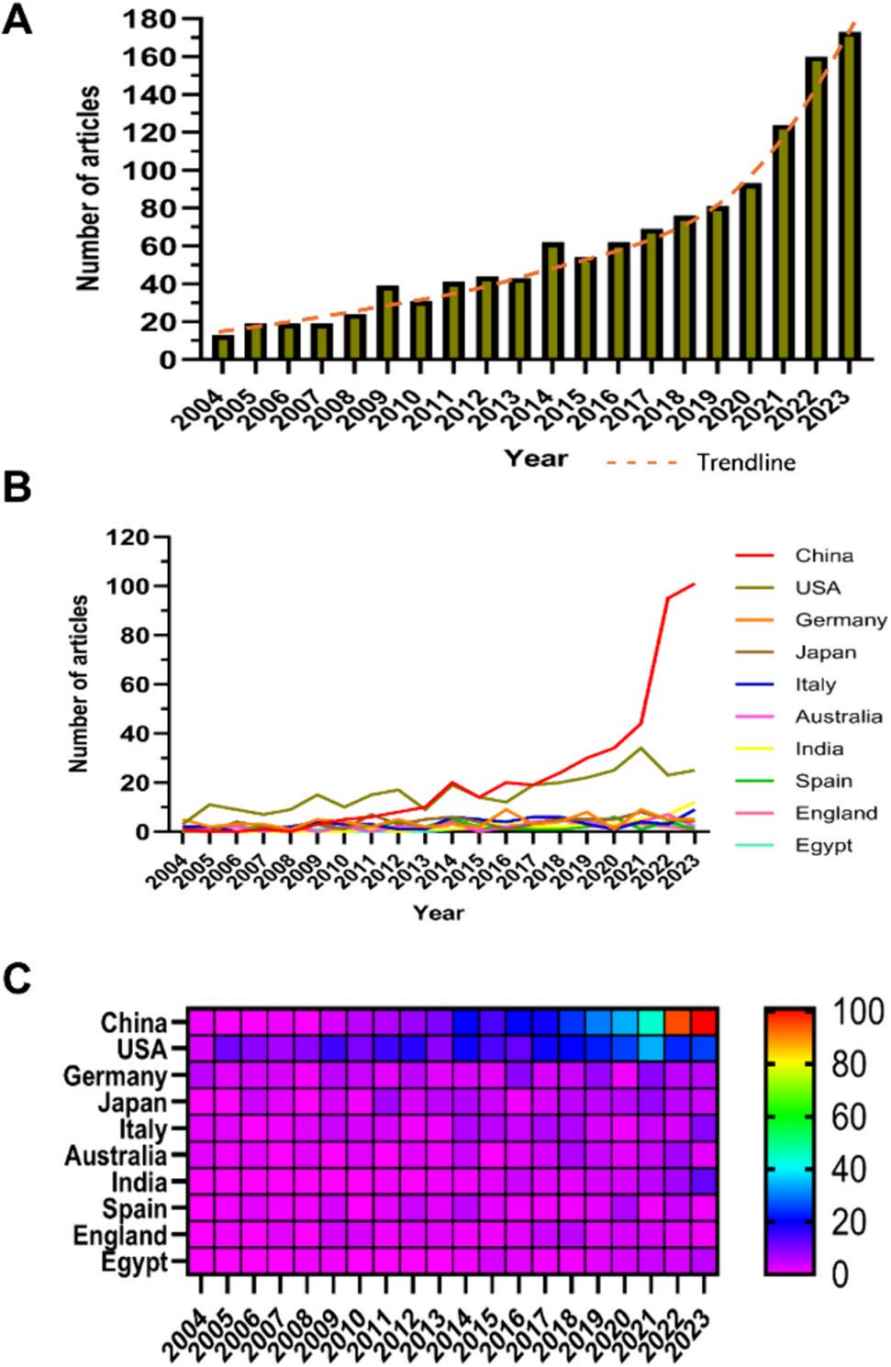
**A** Trend chart of annual publication volume. **B** Line graph of national annual literature publication volume. **C** Heatmap of national annual literature publication volume

Since 2004, there has been a slow increase in the number of publications per year (Fig. 2A). We divided the growth process of publications into three stages (Fig. 2A). The number of publications grew slowly from 2004 to 2008, with fewer than 30 articles per year, suggesting that the field has not received much attention from researchers. The number of publications gradually increased from 2009 to 2013, indicating that the area has gradually entered the researchers' field of vision. After 2014, the number of publications in this field increased rapidly and peaked in 2023, which shows that the field has received widespread attention since 2014.

### Countries and institutions

Research on the role of immunity in DN has been conducted in 81 countries and regions. Figure 2B and C showed the annual publication volume of the top 10 countries during the last two decades, and the top 5 countries in this field were the United States, China, Germany, Italy, and Iran. China accounts for 34.91% of the total number of papers published, far more than any other country (Table 1). Among the top ten countries/regions in terms of the number of published papers, papers from the United States were cited 18,161 times (Table 1), far exceeding all other countries/regions, and its citation/publication ratio (57.11) ranked 5th among all countries/regions, indicating that the quality of its published papers is generally high. China ranked first in the number of publications (435) while ranking second in the number of citations (10,756), and its citation/publication ratio (24.73) ranked at the back of the list, suggesting that the quality of its published papers is generally low.

**Table 1:** Statistics of literature published in the top 10 countries

Rank	Country/region	Article counts	Centrality	Percentage (%)	Citation	Citation per publication
1	China	435	0.10	34.91	10,756	24.73
2	USA	318	0.57	25.52	18,161	57.11
3	Germany	81	0.14	6.50	5791	71.49
4	Japan	71	0.01	5.70	2355	33.17
5	Italy	66	0.16	5.30	2290	34.70
6	Australia	46	0.04	3.69	2910	63.26
7	India	43	0.07	3.45	473	11.00
8	Spain	40	0.09	3.21	3551	88.78
9	England	38	0.14	3.05	3508	92.32
10	Egypt	27	0.03	2.17	358	13.26

**Fig. 3:**
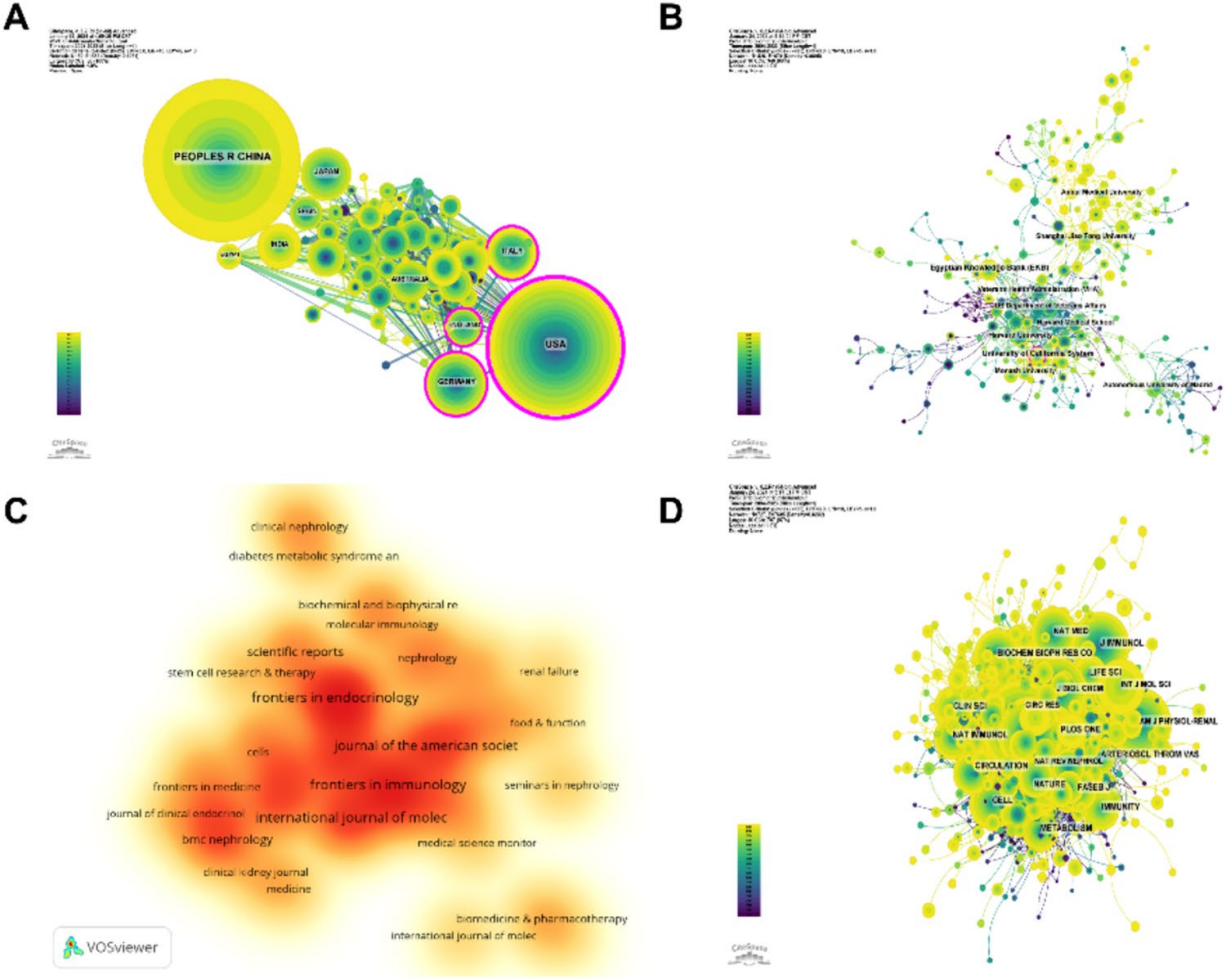
**A** Collaboration network diagram among countries. **B** Collaboration network diagram among institutions. **C** Density chart of published literature in journals. **D** Journal co-cited network map

The collaboration network among countries (Fig. 3A) showed that there was close cooperation between China with the highest production and the United States. The United States had close cooperation with Germany, Italy, and the United Kingdom, while China cooperated more closely with India, Japan, and Egypt. China had not only a large number of publications but also a high citation frequency, indicating that it was currently the leading country in the field. In recent years, countries such as the United States and Japan have seen a rapid increase in the number of publications, which may be related to China's cooperation.

1751 institutions systematically published papers on the role of immunity in DN. Among the top ten institutions in terms of number of publications, five were from the United States, two were from China, one was from Egypt, one was from Australia, and one was from Spain (Table 2, Fig. 3B). The University of California System published the most literature (25 papers, 1062 citations, 42.48 citations per paper). Egyptian Knowledge Bank (EKB) (24 papers, 296 citations, 12.33 citations per paper) ranked second and Harvard University (21 papers, 1411 citations, 67.19 citations per paper) ranked third. After further analysis, we found that domestic and foreign institutions prefer to cooperate with their domestic units, so we call for strengthening cooperation between domestic and foreign institutions and breaking down academic barriers.

**Table 2:** Statistics of literature published in the top 10 institutions

Rank	Institution	Country	Number of studies	Total citations	Average citation
1	University of California System	USA	25	1062	42.48
2	Egyptian Knowledge Bank (EKB)	Egypt	24	296	12.33
3	Harvard University	USA	21	1411	67.19
4	Veterans Health Administration (VHA)	USA	18	662	36.78
5	US Department of Veterans Affairs	USA	18	662	36.78
6	Shanghai Jiao Tong University	China	18	920	51.11
7	Harvard Medical School	USA	17	1118	65.76
8	Anhui Medical University	China	17	394	23.18
9	Monash University	Australia	16	945	59.06
10	Autonomous University of Madrid	Spain	16	921	57.56

### Journals analysis

Table 3 and Fig. 3C showed the top 10 most productive and most cited journals. The *Frontiers in Immunology* (30, 2.41%) was the journal with the most publications in this field, followed by *Frontiers in Endocrinology* (29, 2.33%), *International Journal of Molecular Sciences* (29, 2.33%) and *Journal of the American Society of Nephrology* (24 articles, 1.93%). Among the top ten most prolific journals, *Nature Reviews Nephrology* has the highest impact factor (IF) of 41.5. All these journals were classified as Q1 or Q2.

**Table 3:** Statistics of literature published in the top 10 journals

Rank	Journal	Article counts	Percentage(1246)	IF	Quartile in category
1	Frontiers in Immunology	30	2.41	7.3	Q1
2	Frontiers in Endocrinology	29	2.33	5.2	Q1
3	International Journal of Molecular Sciences	29	2.33	5.6	Q1
4	Journal of the American Society of Nephrology	24	1.93	13.6	Q1
5	Kidney International	20	1.61	19.6	Q1
6	Nephrology Dialysis Transplantation	20	1.61	6.1	Q1
7	American Journal of Physiology-renal Physiology	19	1.52	4.2	Q1
8	Nature Reviews Nephrology	18	1.44	41.5	Q1
9	Plos One	18	1.44	3.7	Q2
10	Scientific Reports	16	1.28	4.6	Q2

Journal impact is determined by its frequency of being co-cited, which indicates whether the journal has a significant impact on the scientific community [36]. According to Fig. 3D and Table 4, the journal with the highest number of co-citations is *Kidney International* (863 citations), followed by the *Journal of the American Society of Nephrology* (827 citations) and *Diabetes* (707). Among the top 10 most co-cited journals, *Kidney International* was cited 863 times and had the highest IF of 19.6. Of the co-cited journals, all were distributed in Q1 or Q2.

**Table 4:** Journal co-citation table

Rank	Cited Journal	Co-Citation	IF(2020)	Quartile in category
1	Kidney International	863	19.6	Q1
2	Journal of the American Society of Nephrology	827	13.6	Q1
3	Diabetes	707	7.7	Q1
4	Nephrology Dialysis Transplantation	614	6.1	Q1
5	Journal of Clinical Investigation	599	15.9	Q1
6	Plos One	574	3.7	Q2
7	Proceedings of the National Academy of Sciences of the United States of America	560	11.1	Q1
8	American Journal of Physiology-renal Physiology	533	4.2	Q1
9	Journal of Immunology	499	4.4	Q2
10	Journal of Biological Chemistry	497	4.8	Q2

### Co-cited references analysis

**Fig. 4:**
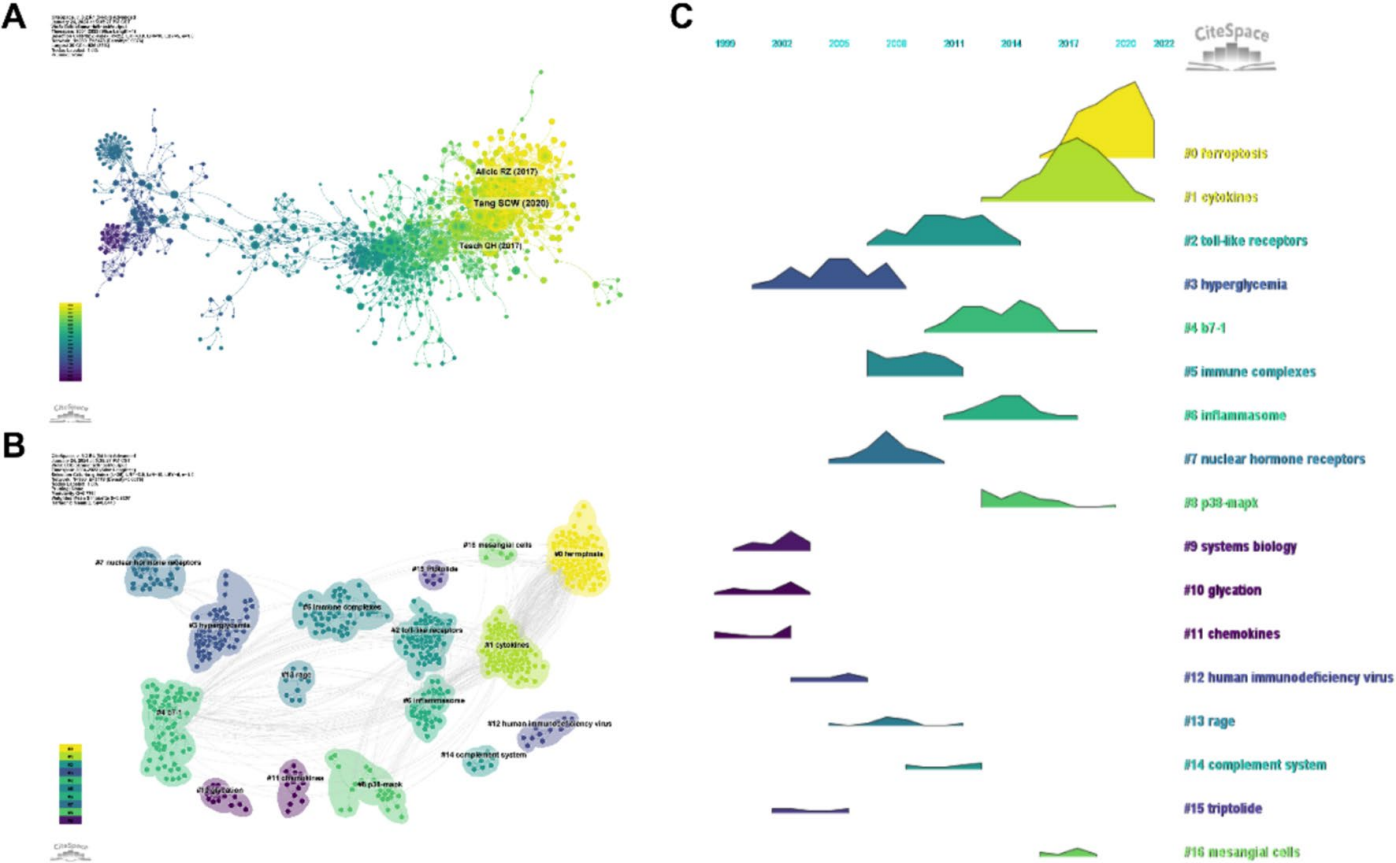
**A** Co-cited references network diagram. **B** Cluster analysis of co-cited references. **C** Timeline ridge plot of co-cited references

With a time slice of one year and a period ranging from 2000 to 2023, the co-cited references network had 960 nodes and 3,478 links (Fig. 4A). Among the top 10 most co-cited references (Table 5), the article titled " Innate immunity in diabetic kidney disease" in *Nature Reviews Nephrology* (IF = 41.5) ranked first as the most co-cited reference, and Escobar- Morreale, Hector F was the first author of the article [37]. Increasing evidence suggests that renal inflammation is an important factor in the pathogenesis and progression of DN and anti-inflammatory therapies may be nephroprotective in DN. In this context, immune cells that activate innate immunity and renal resident cells play a crucial role in triggering and maintaining inflammation. In this article [37], the authors also discussed the mechanisms by which innate immune pathways may contribute to DN and the therapeutic potential of targeting these pathways. For example, Toll-like receptors can induce aseptic tubulointerstitial inflammatory responses through the NF-kB signaling pathway. The NLRP3 inflammasome links the sensing of metabolic stress in diabetic kidneys to the activation of proinflammatory cascades through the induction of IL-1 β and IL-18. Thus, the author suggests that research targeting these innate immune pathways may lead to the development of novel therapies for DN [37].

**Table 5:** References co-cited table

Rank	Title	Journal IF(2021)	Author(s)	Total citations
1	Innate immunity in diabetic kidney disease	Nature Reviews Nephrology (IF = 41.5)	Tang SCW	53
2	Diabetic Kidney Disease Challenges, Progress, and Possibilities	Clinical Journal of the American Society of Nephrology (IF = 9.8)	Alicic RZ	35
3	Diabetic nephropathy—is this an immune disorder?	Clinical Science (IF = 6.0)	Tesch GH	33
4	Innate immunity in diabetes and diabetic nephropathy	Nature Reviews Nephrology (IF = 41.5)	Wada J	29
5	Update on Diabetic Nephropathy: Core Curriculum 2018	American Journal of Kidney Diseases (IF = 13.2)	Umanath K	29
6	Toll-Like Receptor 4 Promotes Tubular Inflammation in Diabetic Nephropathy	Journal of the American Society of Nephrology (IF = 13.6)	Lin M	26
7	Macrophages in diabetic nephropathy in patients with type 2 diabetes	Nephrology Dialysis Transplantation (IF = 6.1)	Klessens CQF	25
8	JAK1/JAK2 inhibition by baricitinib in diabetic kidney disease: results from a Phase 2 randomized controlled clinical trial	Nephrology Dialysis Transplantation (IF = 6.1)	Tuttle KR	24
9	The single-cell transcriptomic landscape of early human diabetic nephropathy	Proceedings of the National Academy of Sciences of the United States of America (IF = 11.1)	Wilson PC	24
10	Inflammation in Diabetic Kidney Disease	Nephron (IF = 2.5)	Perez-Morales, Rosa E	24

We performed co-citation reference clustering and temporal clustering analysis (Fig. 4B and C). We found that hyperglycemia (cluster3), systems biology (cluster9), glycation (cluster10), and chemokines (cluster11) were the early research hotspots. Toll-like receptor (cluster2), immune complexes (cluster5), nuclear hormone receptor (cluster7), human immunodeficiency virus (cluster12), rage (cluster 13), complement system (cluster14), and triptolide (cluster15) were research hotspots in the mid-term. Ferroptosis (cluster0), cytokines (cluster1), b7-1 (cluster4), inflammasome (cluster6), p33-mapk (cluster8), and mesangial cells (cluster16) were the hot topics and trends in this field.

## Keywords analysis

**Fig. 5:**
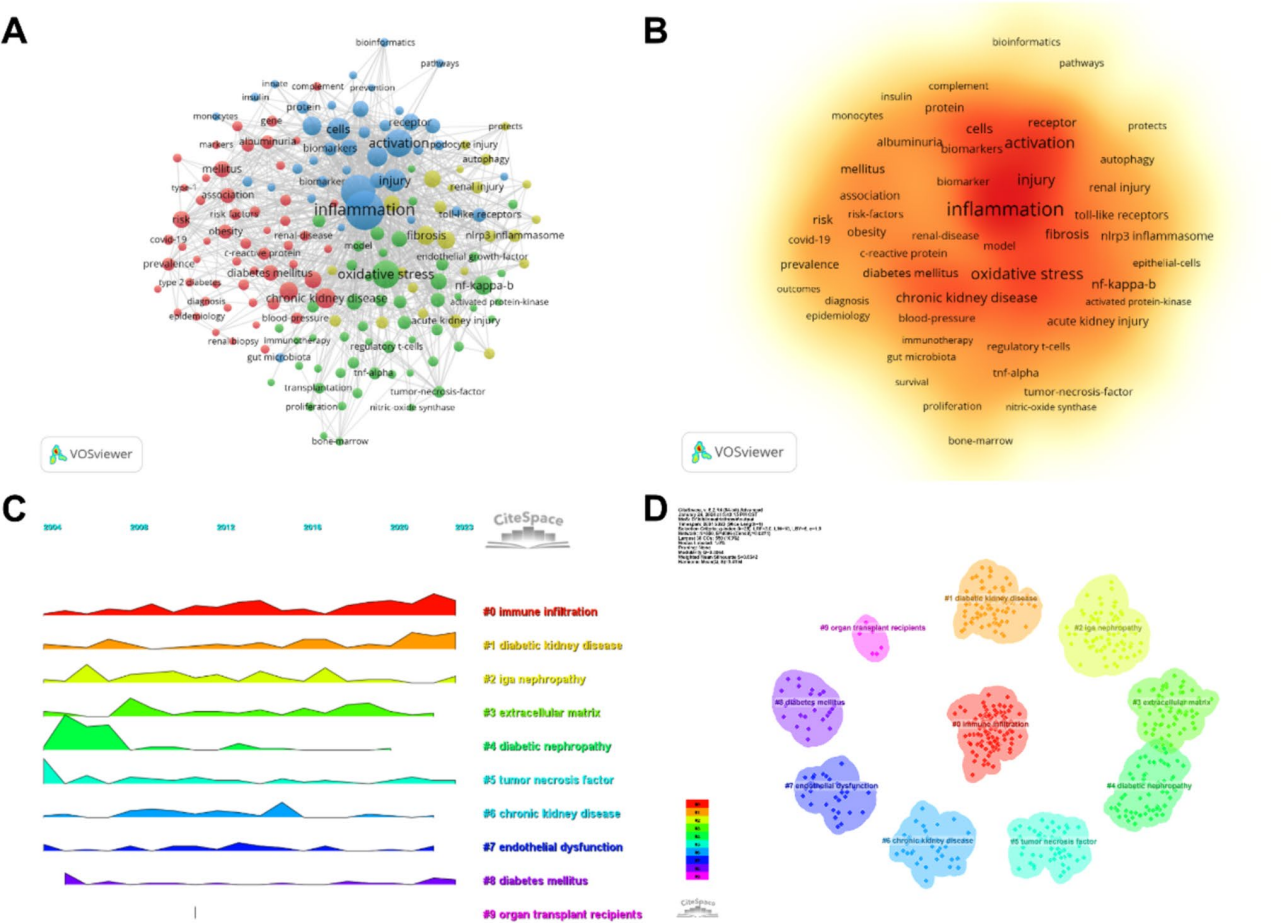
**A** High-frequency keywords network diagram. **B** Density chart of keywords. **C** keywords clustering timeline ridge plot. **D** Cluster analysis of keywords

By analyzing the keywords, we can quickly understand the overview and development direction of this field. Based on the co-occurrence of keywords in VOSwiever software, the most popular keyword was inflammation (269), followed by expression (207), oxidative stress (143), and activation (137) (Table 6, Fig. 5A, B). After removing useless keywords and merging synonyms, We constructed a network containing 172 keywords with at least 12 occurrences, yielding a total of 4 different clusters. Cluster 1 (red) had 54 keywords mainly related to risk factors for DN such as insulin resistance, obesity, diabetes mellitus, genes, metabolic syndrome and so on. Cluster 2 (green) had 53 keywords involving oxidative stress, nitric oxide, and so on.. Cluster 3 (blue) contained 40 keywords, mainly related to immunity and inflammation, such as macrophages, immune complexes, and so on. Cluster 4 (yellow) contained 25 keywords related to pathological changes in DN, such as fibrosis, renal injury, focal segmental glomerulosclerosis, etc. We plotted a cluster diagram through CiteSpace software to visualize the research hotspots over time (Fig. 5C, D).

**Table 6:** List of high-frequency keywords

Rank	Keyword	Counts	Rank	Keyword	Counts
1	Inflammation	269	11	Pathogenesis	67
2	Expression	207	12	Chronic kidney-disease	65
3	Oxidative stress	143	13	Innate immunity	62
4	Activation	137	14	Receptor	57
5	Injury	96	15	Insulin-resistance	56
6	Cells	91	16	Risk	56
7	Mechanisms	78	17	Apoptosis	54
8	Chronic kidney disease	77	18	Mellitus	53
9	nf-kappa-b	70	19	Monocyte chemoattractant protein-1	53
10	Fibrosis	68	20	Macrophages	52

### Burst analysis of co-cited references and keywords

**Fig. 6:**
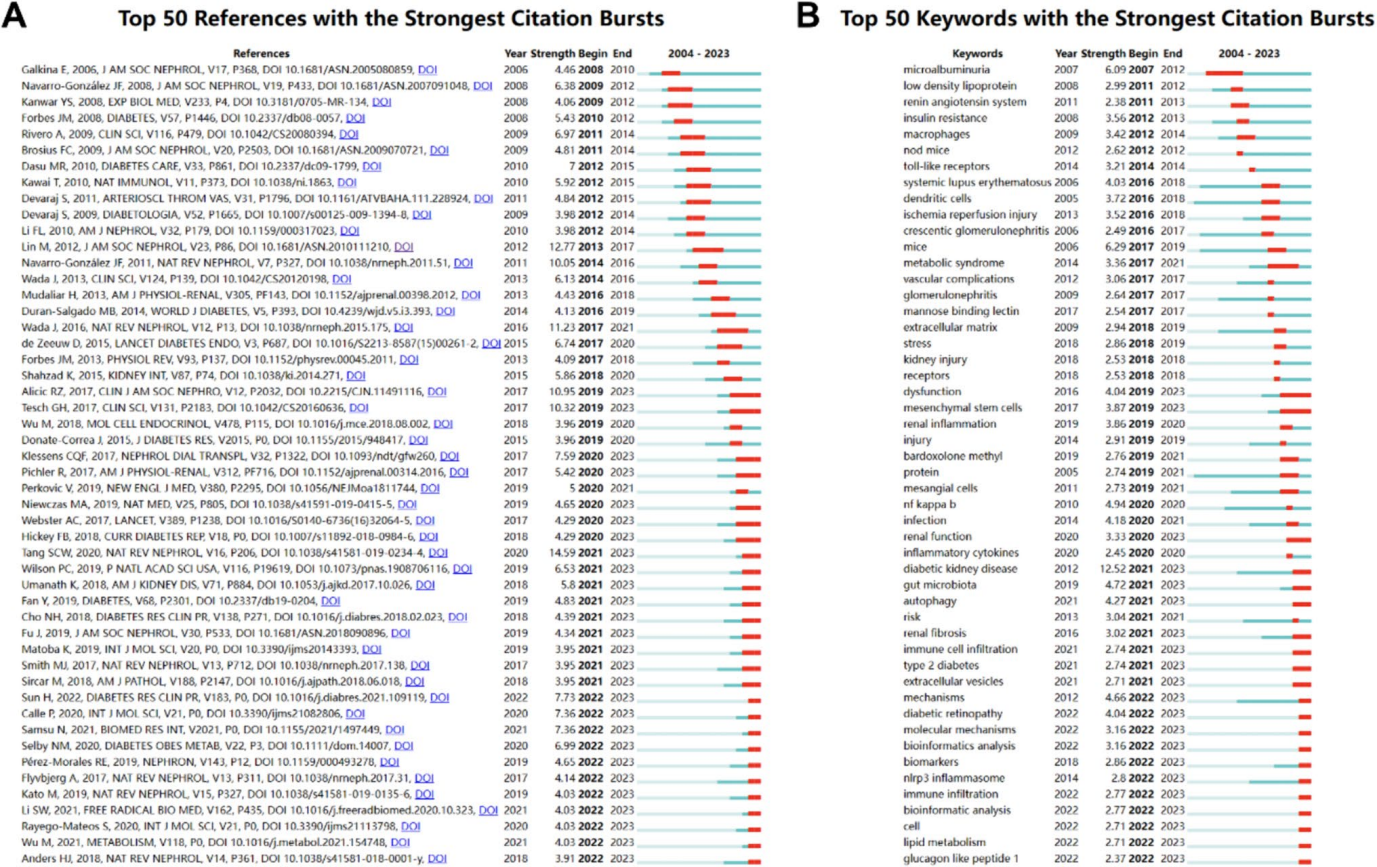
**A** Visualization chart of references with the strongest citation bursts. **B** Visualization chart of keywords with the strongest citation bursts

Using CiteSpace software, we derived the 50 most reliable citation bursts in the field of the role of immunity in DN. One of the most cited (12.77) references was titled " Toll-Like Receptor 4 Promotes Tubular Inflammation in Diabetic Nephropathy" published in the *Journal of the American Society of Nephrology* [38]. The first author of the article was Lin, Miao, which concluded that inflammation was a cause of tubulointerstitial lesions in DN. Toll-like receptors can regulate immune responses and inflammatory diseases, but their function in DN is unclear. In this study [38], the authors found that the expression of TLR4 was increased in the renal tubules of patients with DN and the intensity of its expression was directly related to interstitial macrophage infiltration and hemoglobin A1c levels and inversely associated with the estimated glomerular filtration rate. In vitro experiments demonstrated that hyperglycemia induces TLR4 expression through protein kinase C activation in a time- and dose-dependent manner, leading to up-regulated expression of IL-6 and chemokine (C–C motif) ligand 2 (CCL-2) in human proximal tubular epithelial cells through IκB/NF-κB activation. Overall, these studies indicated that TLR4-mediated pathways may promote tubulointerstitial inflammation in DN [38]. All 50 references were published from 2004 to 2023, suggesting that these papers were frequently cited during the last 20 years. Importantly, 27 of these papers are currently at peak citation (Fig. 6A), which implies that the study of the role of immunity in DN will continue to be of interest in the future.

Among the 786 strongest bursts of keywords in the field, we focused on the 50 keywords with the strongest bursts (Fig. 6B), including microalbuminuria, low density lipoprotein, renin angiotensin system, insulin resistance, macrophages, nod mice, toll-like receptors, systemic lupus erythematosus, dendritic cells, inflammatory cytokines, immune cell infiltration, etc. These keywords represent current research hotspots in the field and possible future research trends.

## Discussion

More and more studies show that the occurrence and progression of DN is closely related to immunity [39, 40]. So far, our study is the first bibliometric review, summary, and foresight in the field. In this study, we analyzed 1,246 pieces of literature from the WoSCC on the role of immunity in DN, and summarized the trends and potential future research hotspots in the field over the past 20 years, hoping to provide insightful perspectives for future studies.

### General information

The number of papers published each year can visually reflect the development status and popularity of a particular field. Our study found that the literature on the role of immunity in DN has tended to increase annually over the past 20 years. It could be roughly categorized into three phases: 2004–2008 was a stagnant period with less than 30 articles per year; 2009–2013 was a slow-growth period with between 30 and 50 articles; and 2014–2023 was a rapid growth period, with a rapid increase in the number of papers per year in the field and reaching a peak in 2023, especially in the last three years when the number of papers per year has exceeded 120. This indicates that the field has attracted sustained academic attention and has become a new hotspot in current research on the DN. Therefore, we venture to speculate that the number of publications in this field will continue to increase.

The number of publications in a research field is an important indicator to assess the level of scientific research of a country, institution or author [41]. Our study found that 81 countries and regions have conducted research on the role of immunity in DN. The top 10 countries published a total of 1,165 publications, with the top 2 countries being China (435) and the United States (318). Obviously, the outstanding contribution of the United States in this field is due to its long-term scientific research foundation, a large number of excellent research talents, abundant research funding, and relatively free academic atmosphere [42]. Interestingly, China, as a developing country, has surpassed the United States to rank first in the number of publications in this field, despite its weak research foundation and late start. This may be related to our large population base, the strategy of rejuvenating the country through science and education, and the fact that we have the largest number of diabetics worldwide [43]. Generally, the total number of citations is a major measure of the quality and attractiveness of an article [44]. Although China ranked first in terms of the number of publications, the total number of citations in the literature was 10,756, and the average number of citations per publication was 24.73, which ranked 8th among the top ten countries in terms of the number of publications. This showed that the quality of the papers published in China was generally low. While the United States had the highest number of citations for publications at 18,161, far outstripping other countries. Notably, the number of papers published in England was 38, but the average number of citations per publication was as high as 92.32, ranking first. This reflected the higher quality of the papers published in England and the United States. Regarding country collaboration, we found close and extensive cooperation between China and the United States. The United States cooperated closely with countries such as Germany, Italy, and England, while China cooperated even more closely with countries such as India, Japan, and Egypt. From the analysis of institutions, among the top ten institutions in terms of the number of publications, five were from the United States and two were from China. Of which, the University of California System, which belongs to the United States, published the most publications (25 papers, 1,062 citations, 42.48 citations per paper). This also reflected the strong research strength of the United States. Shanghai Jiao Tong University from China ranked sixth (18 papers, 920 citations, 51.11 citations per paper), which may be related to Shanghai's location in China's Yangtze River Delta. This may be due to the fact that it is one of the most active regions for economic development, openness, and innovation in China [45]. After further analysis, we found that domestic and foreign institutions preferred to collaborate with organizations within their own countries, and we speculated that this phenomenon might be related to factors such as geographical and cultural differences, varying quality of research methods, and official barriers. If these challenges can be actively addressed, this will facilitate broader international cooperation and innovation, ultimately advancing the field and contributing to scientific progress globally.

Journal source distribution and journal co-citation analysis can provide researchers with valuable information that can help them quickly find the most appropriate target journal when searching the literature or submitting a study [46]. Among the top ten most prolific journals, *Frontiers in Immunology* (30 papers, 2.41%) and *Frontiers in Endocrinology* (29 papers, 2.33%) from the Frontiers publishing platform were ranked first and second, respectively. This indicated that the majority of articles in the field might be considered for publication on this publishing platform. In addition, our study found that *Nature Reviews Nephrology* was the journal with the highest impact factor of 41.5. The most co-cited journal was *Kidney International* (863 citations), followed by the *Journal of the American Society of Nephrology* (827 citations) and *Diabetes* (707 citations). Most of these journals were located in the Q1 region. This showed that studies on the role of immunity in DN were often able to be published in journals of high impact and scholarly value. 

### Research hotspots and trends

To reveal the research frontiers and trends in the field, we performed cluster analysis, co-occurrence analysis, and burst analysis of co-cited references and keywords. The most co-cited reference was entitled " Innate immunity in diabetic kidney disease". This article explored in detail the mechanisms by which innate immune pathways may contribute to DKD and the therapeutic potential of targeting these pathways. Timeline clustering analysis of the co-cited references showed that the latest hot topics in the field involved ferroptosis, cytokines, b7-1, and inflammasome. According to the co-occurrence of keywords, the hottest keywords were mainly inflammation and oxidative stress. Burst analysis of references showed the most cited reference was entitled " Toll-Like Receptor 4 Promotes Tubular Inflammation in Diabetic Nephropathy". The article emphasized the role of TLR4 in promoting tubular inflammation in DN. The 50 keywords with the strongest citation bursts in this field were mainly related to low density lipoprotein, renin angiotensin system, insulin resistance, macrophages, toll-like receptors, dendritic cells, inflammatory cytokines, immune cell infiltration, and so on. Overall, immune-related mechanisms are still the hotspots of DN research, and future research trends may involve the following three aspects: (1) the mechanism of immune-related inflammation involved in DN; (2) the role of ferroptosis in immunity and DN; and (3) the role of lipid metabolism in immunity and DN.

DN was thought to be the result of the interaction of hemodynamic and metabolic factors. Its pathogenesis involved many factors and pathways, among which immune cells and immune-related chronic inflammatory responses played an important role in the occurrence and development of DN [20]. Macrophages are key members of the mononuclear phagocyte system and part of innate immunity. High glucose and late glycosylation end products in the DN environment promote macrophage recruitment, migration, and activation, and activated macrophages release proinflammatory factors, leading to renal injury and fibrosis [47]. Dendritic cells (DCS) are the primary regulators of innate and adaptive immune responses. It can interact with B cells and T cells to manipulate humoral and cellular immune responses [48]. Studies have shown that high glucose can trigger DC maturation and induce a proinflammatory cytokine profile in human DCs, which subsequently mediates tubulointerstitial injury in DN [49, 50]. Besides that, there were many other inflammation-related cytokines aggregated in the renal tissues of patients in the development of DN [18–20], such as chemokines [51], cell adhesion factors [52], growth factors, inflammatory factors, nuclear factors, and so on. However, this mechanism remains controversial and deserves further investigation.

Ferroptosis, a novel mode of cell death induced by iron-dependent oxidative damage, is characterized by lipid peroxidation due to intracellular iron overload and accumulation of reactive oxygen species (ROS) [53]. Recent studies have shown that ferroptosis has been identified as one of the forms of cell death for several immune cells, affecting the immune response. There may be potential interactions between ferroptosis and the immune response in some cases [54]. Iron ions were reabsorbed by renal tubules after glomerular filtration, and renal tubular epithelial cells were extremely active sites for iron ions and ROS. Characteristic changes of ferroptosis, such as iron overload, lipid peroxidation, and mitochondrial alterations, were detected in human renal proximal tubular epithelial cells (HK-2) cultured in high glucose [55]. It was found that ferroptosis was closely associated with renal tubular cell death in diabetic conditions [56]. These findings have also been used to explain the action mechanisms of drugs which may be potentially valuable in the treatment of DN. For example, Ghanim et al.'s study found that dagliflozin was effective in reducing serum iron-regulatory protein levels and improving iron metabolism disorders in patients with type 2 diabetes mellitus [57]. Huang et al. observed that liraglutide alleviated glomerular extracellular matrix accumulation and renal injury in DN by enhancing Wnt/β-catenin signaling [58]. Therefore, an in-depth exploration of the mechanism of ferroptosis in immunity and DN could provide new therapeutic ideas and potential drug targets for DN patients.

Studies have shown that lipid metabolism could regulate the differentiation and function of immune cells [59]. T helper cells secreting IL-17 play a pathogenic role in a variety of inflammatory and autoimmune diseases, and its development requires endogenous fatty acid synthesis [60]. Different types of macrophages have different requirements for fatty acid synthesis and catabolism. Factors that promote M1 macrophages induce fatty acid synthesis, whereas anti-inflammatory signals that favor M2 macrophages drive fatty acid oxidation [61]. Conversely, immune cells are an important part of the lipid microenvironment, influencing local and systemic lipid metabolism [62]. Renal lipid homeostasis has received increasing attention in recent years [63]. Altered fatty acid and cholesterol metabolism were recognized as crucial pathways for renal lipid accumulation, inflammation, oxidative stress, and fibrosis [64]. Oxidized low-density lipoprotein (ox-LDL) may promote the development of DN by damaging renal endothelial, thylakoid, and tubular cells through lipid metabolic pathways, inflammatory damage, and hemodynamic factors [65]. In addition, abnormal renal lipid metabolism was found to be present in the kidneys of mice with DN and high glucose-induced renal podocytes [66]. Thus, podocyte lipid accumulation has been considered a potential therapeutic target for DN. Looking forward, understanding more regulatory details of lipid metabolism involved in immunity and DN will provide more valuable clues for the study and treatment of DN.

### Limitations

Although, we were able to gain a detailed view of the research evolution and global trends in the relationship between immunity and DN using visual analysis tools such as CiteSpace and VOSviewer. However, this study also has some limitations. First, we only retrieved the WoSCC database in this study, and there were many other noteworthy databases that have not yet been searched, such as the Scopus, PubMed, and Embase databases. However, considering that the WoSCC database is one of the most used and comprehensive global databases [67–71], it is sufficient to reflect the general trend in this research field. Second, we only analyzed studies published in English, which could lead to the fact that some non-English studies could be missed due to language limitations. Finally, considering the presence of near-synonyms, abbreviations, and full names, the keyword bursts may differ from the actual results. Therefore, the results need to be interpreted with caution.

## Conclusion

In conclusion, immunization plays an important role in the development of DN. The number of publications related to immunity and DN has increased annually over the past 20 years, with a significant increase especially in the last 3 years, indicating that researchers are increasingly interested in this field. China and the United States are the two countries with the highest number of publications and close collaboration. The University of California System is the most prolific institution. *Frontiers in Immunology* is the journal with the most publications in the field. Future directions may involve immune-related inflammation, ferroptosis, and lipid metabolism. This study summarized the current situation and global trends in research on the role of immunity in DN and provided valuable suggestions and ideas for future research.

## Data Availability

All original contributions are included in the manuscript.
